# From Phosphorus Nutrition to Starch Functionality: Effects of Phosphorus Fertilization on Sorghum Starch Biosynthesis, Multiscale Structure, and Processing Properties

**DOI:** 10.3390/foods15203651

**Published:** 2026-10-15

**Authors:** Baizhi Chen, Linlin Yang, Fulai Ke, Kai Zhu

**Affiliations:** Sorghum Research Institute, Liaoning Academy of Agricultural Sciences, Shenyang 110161, China

**Keywords:** sorghum starch, phosphorus fertilization, starch structure, starch biosynthesis enzymes, gelatinization properties

## Abstract

Phosphorus fertilization can regulate starch accumulation and quality in cereal grains, but its effects on sorghum starch remain insufficiently understood. This study investigated sorghum cultivar Liaoza 82 under four phosphorus levels: 0, 40, 80, and 160 kg P_2_O_5_ ha^−1^. Phosphorus application significantly affected starch accumulation and the activities of starch biosynthesis-related enzymes, including AGPase, GBSS, SSS, and SBE. Moderate phosphorus, particularly 80 kg P_2_O_5_ ha^−1^, increased amylose content, granule size, molecular weight, amylopectin branching degree, and medium-to-long amylopectin chains, whereas the highest phosphorus treatment weakened these effects and caused granule surface depressions. Although the A-type crystalline pattern was unchanged, phosphorus modified crystallinity, short-range order, thermal properties, solubility, swelling power, and pasting behavior. Within the phosphorus range investigated, P2 produced a favorable combination of selected structural and thermal characteristics, including lower crystalline order and lower gelatinization temperature and enthalpy, whereas some pasting parameters were highest under P3. These findings indicate that phosphorus fertilization alters sorghum starch structure and functionality, with the response depending on the specific starch characteristic evaluated.

## 1. Introduction

*Sorghum bicolor* (L.) Moench is a drought- and salinity-tolerant C4 cereal that is widely cultivated in semi-arid and marginal environments [1,2]. Traditionally valued as a staple food and feed resource, sorghum is now increasingly recognized as a promising industrial starch crop [3]. Sorghum grains contain approximately 65–80% starch, and this carbohydrate reserve exhibits distinct physicochemical properties, such as a characteristic amylose-to-amylopectin ratio, crystalline organization, chain length distribution of amylopectin branches, and unique gelatinization behavior [4]. These features render sorghum starch highly suitable for non-food applications, including bioethanol production, biodegradable polymers, starch-based adhesives and films, and pharmaceutical excipients [5,6,7]. With global demand for renewable, bio-based raw materials continuing to rise, integrating sorghum starch into industrial value chains has become a subject of considerable scientific and commercial interest.

The structural and functional quality of starch is strongly shaped by both genetic factors and agronomic practices [8]. Among essential nutrients, phosphorus (P) plays a pivotal role because of its direct involvement in energy metabolism and carbon assimilation pathways [9]. Phosphorus is a structural component of ATP and other phosphorylated intermediates, which are indispensable for the operation of starch biosynthetic enzymes such as ADP-glucose pyrophosphorylase (AGPase), starch synthases (SS), and starch branching enzymes (SBEs) [10,11]. Adequate P availability ensures efficient generation and utilization of ADP-glucose, the immediate precursor for starch polymerization, while deficiency restricts carbohydrate conversion into starch, resulting in reduced accumulation and altered molecular properties [12]. In sorghum, this implies that phosphorus fertilization has the potential to directly modulate starch content, granule morphology, and molecular organization.

Previous studies in wheat and mung bean have shown that phosphorus availability significantly affects grain composition and starch characteristics [8,12]. Phosphorus deficiency often leads to smaller starch granules, lower crystallinity, and impaired gelatinization behavior, which can reduce processing quality [13]. Conversely, sufficient P supply has been associated with improved starch accumulation, modified amylose-to-amylopectin ratios, and favorable pasting properties that enhance industrial functionality [8]. However, most previous studies have focused on individual starch traits or on crops other than sorghum, and relatively little is known about how phosphorus fertilization simultaneously affects starch accumulation, biosynthesis-related enzyme activities, molecular structure, granular characteristics, crystalline organization, and functional properties in sorghum. In particular, the relationships among these multilevel starch traits under different phosphorus inputs remain insufficiently characterized. While these effects have been well documented in other cereals, systematic evaluations in sorghum remain limited. This knowledge gap restricts the ability to fine-tune fertilization strategies for optimizing sorghum starch yield and quality, especially when targeting non-food, industrial applications.

Understanding how phosphorus fertilization influences sorghum starch is therefore critical for advancing its role as both a staple crop and an industrial raw material. Linking phosphorus management to starch structure and function could provide evidence-based guidelines for agronomic practices that improve sorghum productivity and tailor starch properties to meet industrial processing requirements. The present study was designed to investigate the effects of different phosphorus fertilization regimes on the synthesis, structural features, and functional properties of sorghum starch. We analyzed multi-scale starch traits, including amylose content, chain length distribution, crystallinity, and thermal behavior, under contrasting phosphorus inputs. By integrating field fertilization management with detailed starch structural analysis, this study aims to provide new insights into how phosphorus fertilization can be leveraged to optimize sorghum grain composition and reinforce its potential as a sustainable bio-based material crop.

## 2. Materials and Methods

### 2.1. Plant Materials and Experimental Design

The sorghum (*Sorghum bicolor* L. Moench) cultivar Liaoza 82 (LZ82) was provided and maintained by the Sorghum Research Institute of the Liaoning Academy of Agricultural Sciences. Field experiments were conducted at the experimental farm of the Liaoning Academy of Agricultural Sciences, Shenyang, Liaoning Province, China. In 2023, the site had a mean annual temperature of 9.7 °C and an average annual precipitation of 651.7 mm. The initial soil had a pH of 7.45 and contained 16.53 g kg^−1^ organic matter, 6.81 mg kg^−1^ available phosphorus, 100.37 mg kg^−1^ available potassium, 1.31 mg kg^−1^ NH_4_^+^-N, and 0.69 mg kg^−1^ NO_3_^−^-N. Plant-available soil phosphorus was determined according to the Chinese agricultural industry standard NY/T 1121.7–2014 [14], using 0.5 mol L^−1^ NaHCO_3_ extraction followed by molybdenum–antimony colorimetric determination. The total nitrogen, phosphorus, and potassium contents were 0.083%, 0.527 g kg^−1^, and 2.747 g kg^−1^, respectively. The experiment was arranged in a randomized complete block design with four phosphorus (P) fertilization treatments: 0 (P0), 40 (P1), 80 (P2), and 160 (P3) kg P_2_O_5_ ha^−1^. Three independent blocks were established, and the four P treatments were randomly assigned to separate plots within each block, resulting in three independent field replicates per treatment and a total of 12 experimental plots. Each plot constituted an independent experimental unit.

Each experimental plot consisted of 10 rows, with a row length of 10 m, a row spacing of 0.50 m, and a plant spacing of 0.17 m, corresponding to an area of approximately 50 m^2^ per plot. A 2 m wide buffer alley was maintained between adjacent plots. For sample collection, three plants were sampled from each plot and combined to form one composite biological sample for that plot. Samples from different plots were kept separate throughout sample preparation and subsequent analyses. Thus, the three plots within each P treatment represented three independent biological replicates (*n* = 3).

Phosphorus fertilizer was applied as single superphosphate (16% P_2_O_5_). Nitrogen and potassium fertilizers were applied as basal fertilizers at rates of 150 kg N ha^−1^ as urea (46% N) and 90 kg K_2_O ha^−1^ as potassium chloride (60% K_2_O), respectively, in accordance with local agronomic practices. No additional fertilizers were applied during crop growth. Sorghum was sown on 15 May 2023 and harvested on 25 September 2023, and standard local field management practices were applied consistently across all experimental plots.

### 2.2. Starch Content and Amylose Content

Sorghum grains were sampled at multiple stages after flowering and oven-dried to a constant weight to ensure reliable determination of grain constituents. Starch content in sorghum flour was quantified following enzymatic hydrolysis and subsequent measurement using the glucose oxidase–peroxidase (GOPOD) assay [15,16]. Amylose content (AC) was determined using a dual-wavelength spectrophotometric method as described by [17], while amylopectin content was calculated as the difference between total starch and amylose content [18].

### 2.3. Enzyme Activity Measurements

Grain samples of sorghum were obtained at defined developmental stages from plants subjected to different treatments. Sampling was performed every seven days starting at 7 days post anthesis and continuing until 28 days post anthesis, with all collections completed during the morning period (09:00). Immediately after harvest, grains were snap-frozen in liquid nitrogen and preserved at −80 °C until biochemical analyses were conducted. For enzyme extraction, frozen grains were finely ground under chilled conditions in a 0.1 mol·L^−1^ Tris-based buffer adjusted to the appropriate pH with NaOH. The resulting extracts were clarified by centrifugation at 4 °C for 30 min at 8000× *g*. The supernatants were used for the determination of SBE, SSS, and AGPase activities, whereas the starch-granule-containing precipitates were retained and resuspended in the corresponding extraction buffer for GBSS activity determination. Activities of enzymes involved in starch biosynthesis, including granule-bound starch synthase (GBSS), starch branching enzyme (SBE), soluble starch synthase (SSS), and ADP-glucose pyrophosphorylase (AGPase), were quantified using commercially available assay kits (Solarbio, Beijing, China).

### 2.4. Starch Isolation

Sorghum starch was extracted from mature grains using an alkaline steeping method with slight modifications [8]. Briefly, dehulled sorghum kernels were ground into flour and soaked in 3 g L^−1^ NaOH solution at 35 °C for 12 h to remove proteins and other non-starch components. The resulting slurry was successively filtered through 100- and 200-mesh sieves, followed by centrifugation at 3000× *g* for 10 min. After the supernatant was removed, the upper yellow layer was carefully scraped off, and the remaining white starch sediment was collected. The starch pellet was washed several times with distilled water, neutralized with 0.1 mol L^−1^ HCl, and then centrifuged again. Finally, the purified starch was dried at 40 °C and passed through a 100-mesh sieve for subsequent analyses.

### 2.5. Scanning Electron Microscopy

The morphology of sorghum starch granules was observed using scanning electron microscopy. Prior to observation, the extracted starch was ground and sieved through a 100-mesh screen. Approximately 0.1 g of starch was placed in a 2 mL centrifuge tube, mixed with 1 mL of 4% SDS washing buffer, and vortexed thoroughly. The suspension was centrifuged at 10,000× *g* for 1 min, and the supernatant was discarded. This washing procedure was repeated twice. The starch pellet was then washed five times with deionized water under the same centrifugation conditions. After washing, 1 mL of absolute ethanol was added and mixed evenly to obtain a starch suspension [16].

Approximately 200 μL of the suspension was dropped onto conductive adhesive attached to a copper sample stage and spread evenly. The samples were dried overnight at 37 °C, coated with gold using an ion sputter coater, and observed with a Zeiss Merlin Compact scanning electron microscope (Carl Zeiss, Oberkochen, Germany). Images were captured at an accelerating voltage of 3.00 kV, a working distance of 8.5 mm, and a magnification of 2000× using a secondary electron detector.

### 2.6. Starch Granule Size Analysis

The particle size distribution of sorghum starch was determined using a Mastersizer 3000 laser diffraction particle size analyzer (Malvern Instruments Ltd., Worcestershire, UK). For each measurement, 100 mg of starch was dispersed in 1 mL of 75% ethanol and treated by ultrasonication to ensure uniform suspension. The measurement range was set from 0.1 to 3500 μm. The number-, volume-, and surface-weighted mean diameters, as well as d(0.1), d(0.5), and d(0.9), were calculated according to the previously reported method [19].

### 2.7. Determination of Starch Molecular Weight

The molecular weight distribution of starch was analyzed using size-exclusion chromatography coupled with multi-angle laser light scattering and refractive index detection. Starch samples were first dissolved by adding 5 mg of starch to 5 mL of 0.5% LiBr/DMSO solution and heating at 80 °C for 3 h. The dissolved samples were then separated on a series of Ohpak columns at 60 °C. The mobile phase was 0.5% LiBr/DMSO, delivered at a flow rate of 0.3 mL min^−1^ under isocratic elution. Data were analyzed using ASTRA 6.1 software to obtain number-average molecular weight (Mn), weight-average molecular weight (Mw), Z-average molecular weight (Mz), root-mean-square radius of gyration (Rz), and polydispersity index (PDI) [16].

### 2.8. Chain Length Distribution and Branching Degree Analysis

The chain length distribution of amylopectin was determined by HPAEC-PAD using a Dionex ICS5000 system (Thermo Fisher Scientific, Waltham, MA, USA). Purified starch (10 mg) was gelatinized in 5 mL of water in a boiling water bath for 60 min, followed by addition of 10 μL of 2% (*w*/*v*) sodium azide, 50 μL of 0.6 M sodium acetate buffer (pH 4.4), and 10 μL of isoamylase (1400 U; Sigma-Aldrich, St. Louis, MO, USA). The mixture was incubated at 37 °C for 24 h. Isoamylase was then inactivated by heating the mixture in a boiling water bath for 10 min. After cooling, the debranched glucans were reduced with 0.5% (*w*/*v*) sodium borohydride for 20 h. After drying, the sample was dissolved in NaOH and analyzed on a CarboPac PA-200 column (250 × 4.0 mm) at a flow rate of 0.4 mL min^−1^ with an injection volume of 5 μL. The elution gradient was performed as previously described [16].

The branching degree of starch was determined by proton nuclear magnetic resonance (^1^H NMR) spectroscopy [8]. Approximately 5 mg of purified starch was dissolved in 1 mL of DMSO-d_6_ by heating at 80 °C overnight. After centrifugation at 12,000× *g* for 10 min, the supernatant was transferred into an NMR tube. ^1^H NMR spectra were recorded on a Bruker BioSpin NMR spectrometer (Bruker BioSpin GmbH, Rheinstetten, Germany) operating at 500.23 MHz with 32 scans. The spectra were processed using MestReNova 15.1.0 software. The characteristic signal regions corresponding to α-1,6 and α-1,4 glycosidic linkages were selected according to their respective chemical shifts, and the corresponding peak areas were integrated. The branching degree (DB) was calculated as follows:

DB (%) = A/(A + B) × 100

where A represents the integrated peak area of the α-1,6 glycosidic linkage and B represents the integrated peak area of the α-1,4 glycosidic linkage.

### 2.9. X-Ray Diffraction and Fourier-Transform Infrared Spectroscopy

The crystalline structure of sorghum starch was characterized by X-ray diffraction using a D/Max2550VB+/PC diffractometer (Rigaku Corporation, Tokyo, Japan). Samples were scanned with Cu Kα radiation (λ = 0.15406 nm) at 40 kV and 40 mA. The scanning range was 4–60° 2θ, with a step size of 0.02° and a scanning speed of 4° min^−1^. The crystalline pattern and relative crystallinity were analyzed using MDI Jade 5.0 software [16]. Relative crystallinity was determined from the XRD profiles after baseline correction by separating the crystalline and amorphous contributions. The integrated areas of the crystalline region (Ac) and amorphous region (Aa) were obtained using the same area-integration procedure for all samples, and relative crystallinity was calculated as Ac/(Ac + Aa) × 100%. The same baseline correction and data processing parameters were applied consistently to all samples. No additional peak-fitting procedure was applied.

Short-range ordered structure was evaluated by Fourier-transform infrared spectroscopy using a Nicolet iZ-10 spectrometer (Thermo Fisher Scientific, Waltham, MA, USA). Dried starch samples were thoroughly mixed with KBr and compressed into pellets. Spectra were collected from 4000 to 400 cm^−1^ at a resolution of 4 cm^−1^, with 32 scans recorded for each sample [16]. The spectral region of 1200–900 cm^−1^ was used for analysis of starch short-range molecular order. The absorbance intensities at approximately 1047 and 1022 cm^−1^ were obtained from the processed spectra, and the A1047/A1022 ratio was calculated as an empirical spectroscopic indicator of short-range molecular order. The same spectral processing procedure was applied to all samples.

### 2.10. Water Solubility and Swelling Power

Water solubility and swelling power of sorghum starch were determined with minor modifications [8]. Approximately 0.1 g of starch was dispersed in 10 mL of deionized water and heated in a water bath at 95 °C for 60 min, with gentle shaking every 10 min. After cooling to room temperature in an ice bath, the samples were centrifuged at 8000× *g* for 20 min. The supernatant was dried at 80 °C to constant weight for determination of water solubility, while swelling power was calculated from the mass of the dried starch sediment.

### 2.11. Pasting Properties

Pasting properties of sorghum starch were measured using a Rapid Visco Analyzer (Techmaster, Newport Scientific Pty Ltd, Warriewood, Australia) according to GB/T 24852-2010 [20]. A starch suspension was prepared using 3.00 g of starch and 25.0 mL of distilled water, with the sample and water amounts corrected to a 12% moisture basis. The suspension was initially stirred at 960 rpm for 10 s and then maintained at 160 rpm throughout the measurement. The sample was held at 50 °C for 1 min, heated to 95 °C at 12 °C min^−1^, maintained at 95 °C for 2.5 min, cooled to 50 °C at 12 °C min^−1^, and held at 50 °C for 1.4 min. Peak viscosity, trough viscosity, breakdown, final viscosity, setback, and pasting time were recorded.

### 2.12. Thermal Properties

Thermal properties of sorghum starch were analyzed using a DSC system (Q2000, TA Instruments, New Castle, DE, USA) [8]. Starch sample (3 mg) and distilled water (6 μL) were sealed in an aluminum pan and equilibrated at 4 °C overnight. The sample was heated from 40 to 100 °C at 10 °C min^−1^, with an empty pan as reference. Onset temperature, peak temperature, conclusion temperature, and gelatinization enthalpy were obtained from the thermogram.

### 2.13. Statistical Analysis

Data are expressed as the mean ± standard deviation of three independent field replicates (*n* = 3). For starch content and starch biosynthesis-related enzyme activities measured at different developmental stages, analysis of variance was performed using a model including phosphorus treatment (P), developmental stage (Stage), the P × Stage interaction, and block. Phosphorus treatment and developmental stage were treated as fixed factors, whereas block was included as a blocking factor to account for the randomized complete block design. Where appropriate, differences among phosphorus treatments within each developmental stage were further evaluated using Duncan’s multiple range test at *p* < 0.05. For other starch structural and functional properties measured at maturity, phosphorus treatment effects were evaluated using analysis of variance with phosphorus treatment as the fixed factor and block included as a blocking factor, followed by Duncan’s multiple range test at *p* < 0.05 when a significant treatment effect was detected. Differences among treatments were analyzed by one-way analysis of variance, followed by Duncan’s multiple range test using SPSS 23.0, with significance set at *p* < 0.05. Two-way ANOVA, Pearson correlation analysis and quadratic regression were performed in R (version 4.6.1). Response patterns were classified as inverted-U-shaped, U-shaped, linearly positive, or linearly negative according to the regression coefficients and significance. The Benjamini–Hochberg method was used for multiple-testing correction. Figures were generated using the linkET (version 0.1.0) and ggplot2 (version 4.0.3) packages.

## 3. Results and Discussion

### 3.1. Effects of Phosphorus Fertilization on Starch Content

**Figure 1:**
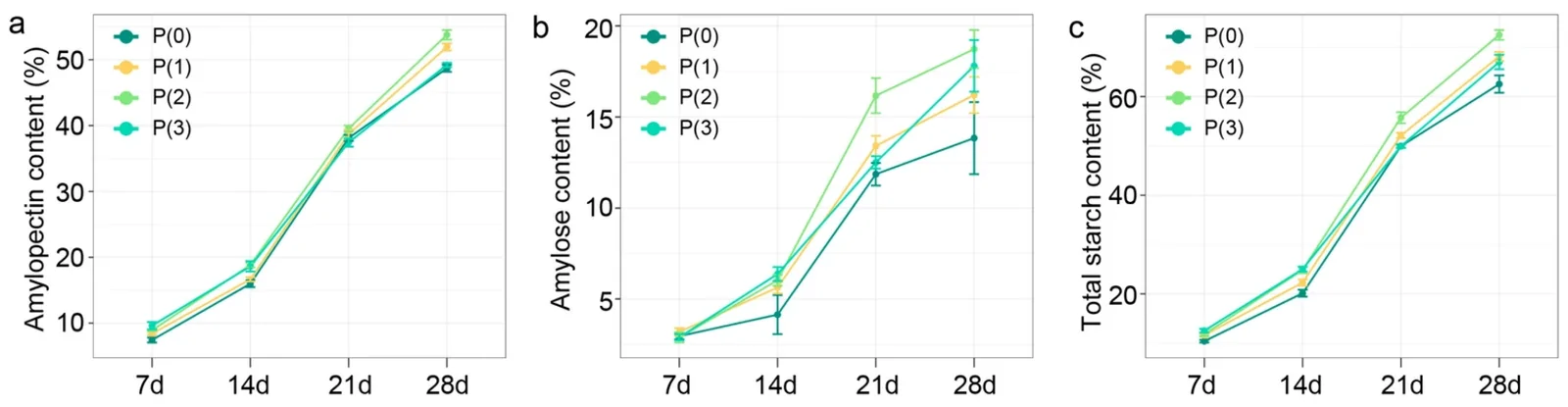
Effects of different phosphorus fertilizer treatments on starch content in sorghum. (**a**–**c**) represent amylopectin, amylose, and total starch content, respectively.

Although the effects of phosphorus fertilization on starch content remain somewhat controversial [21,22], our results clearly indicate that the application of phosphorus promotes starch accumulation (Figure 1). On the one hand, phosphorus enhances photosynthetic efficiency, thereby increasing the supply of assimilates and accelerating dry matter accumulation [23]. Previous studies have suggested that phosphorus availability may regulate the expression of starch biosynthesis-related genes [12]. These findings suggest that phosphorus fertilization is associated with altered starch accumulation patterns during grain development, while the underlying regulatory mechanisms require further investigation. Interestingly, while high phosphorus input (P3) accelerated starch accumulation during the early grain-filling stage, its stimulatory effect diminished after 21 days, and the final starch content was only slightly higher than that of the no-phosphorus control (P0). Stage-specific comparisons confirmed that the effects of phosphorus treatment differed across grain development, with significant differences among treatments becoming particularly evident for amylopectin, amylose, and total starch at the middle and late grain-filling stages (Table S1). Excessive fertilization has been reported to disrupt the balance between vegetative and reproductive growth, negatively affect nutrient accumulation, and even suppress starch biosynthesis, ultimately reducing starch content [8]. Amylose also exhibited a clear response to phosphorus levels during grain development (Figure 1b). Further research is therefore required to elucidate the physiological and molecular mechanisms by which phosphorus fertilization influences starch biosynthesis in crops. Analysis of variance accounting for the randomized complete block design revealed significant effects of phosphorus treatment, developmental stage, and their interaction on total starch, amylopectin, and amylose contents (*p* < 0.001; Table S2), indicating that the effects of phosphorus fertilization varied across grain developmental stages.

### 3.2. Phosphorus Fertilization Affects Starch Accumulation by Regulating Key Enzyme Activities

Starch biosynthesis is coordinated by multiple enzymes, among which ADP-glucose pyrophosphorylase (AGPase), granule-bound starch synthase (GBSS), soluble starch synthase (SSS), and starch branching enzyme (SBE) are considered the key rate-limiting enzymes [24]. Analysis of variance accounting for block effects revealed significant main effects of phosphorus treatment and developmental stage on the activities of AGPase, GBSS, SSS, and SBE (*p* < 0.001), whereas the phosphorus treatment × developmental stage interactions were not significant for any of the four enzymes (*p* > 0.05; Table S3). Block effects were also not significant for any of the four enzymes (*p* > 0.05). These results indicate that enzyme activities varied significantly with phosphorus treatment and grain developmental stage, while no significant P × Stage interaction was detected.

**Figure 2:**
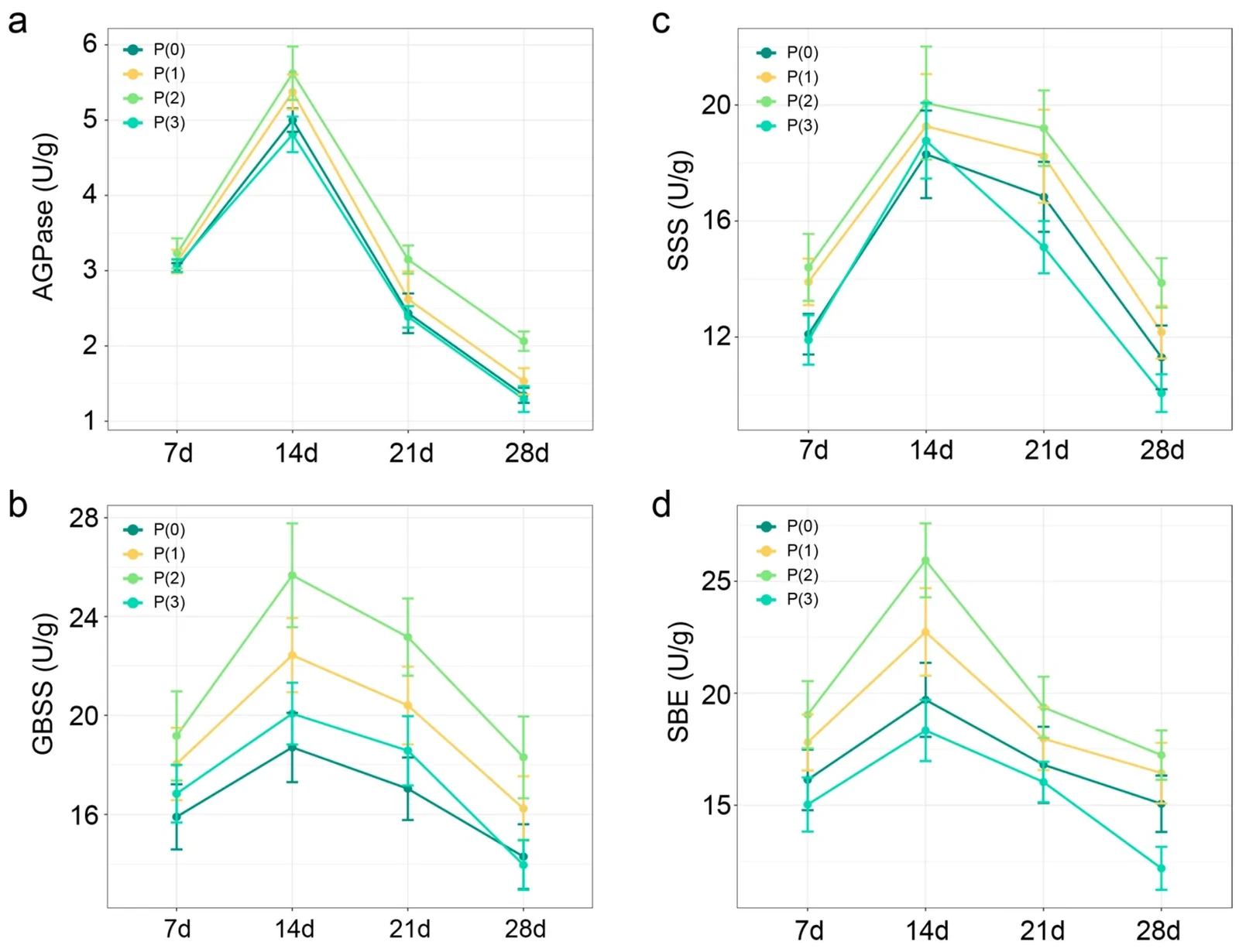
The effects of different phosphorus fertilizer treatments on the activities of key enzymes involved in starch biosynthesis. (**a**) ADP-glucose pyrophosphorylase (AGPase); (**b**) granule-bound starch synthase (GBSS); (**c**) soluble starch synthase (SSS); and (**d**) starch branching enzyme (SBE).

Compared with the no-phosphorus control (P0), moderate phosphorus application (P2) generally resulted in higher activities of AGPase, GBSS, SSS, and SBE, although the significance of treatment differences varied among developmental stages (Table S4). In contrast, the highest phosphorus treatment (P3) did not consistently further increase enzyme activities, indicating that excessive phosphorus supply provided no additional enhancement. Enzyme activities also varied markedly across developmental stages, consistent with the dynamic changes in starch accumulation during grain development. GBSS activity showed a clear response to phosphorus treatment, and the relatively high GBSS activity under P2 was accompanied by higher amylose accumulation (Figure 2b). This suggests an association between GBSS activity and amylose accumulation. Overall, phosphorus fertilization significantly altered starch biosynthesis-related enzyme activities and may contribute to changes in starch accumulation and composition.

### 3.3. Effects of Phosphorus Supply on Starch Granule Morphology and Size

**Figure 3:**
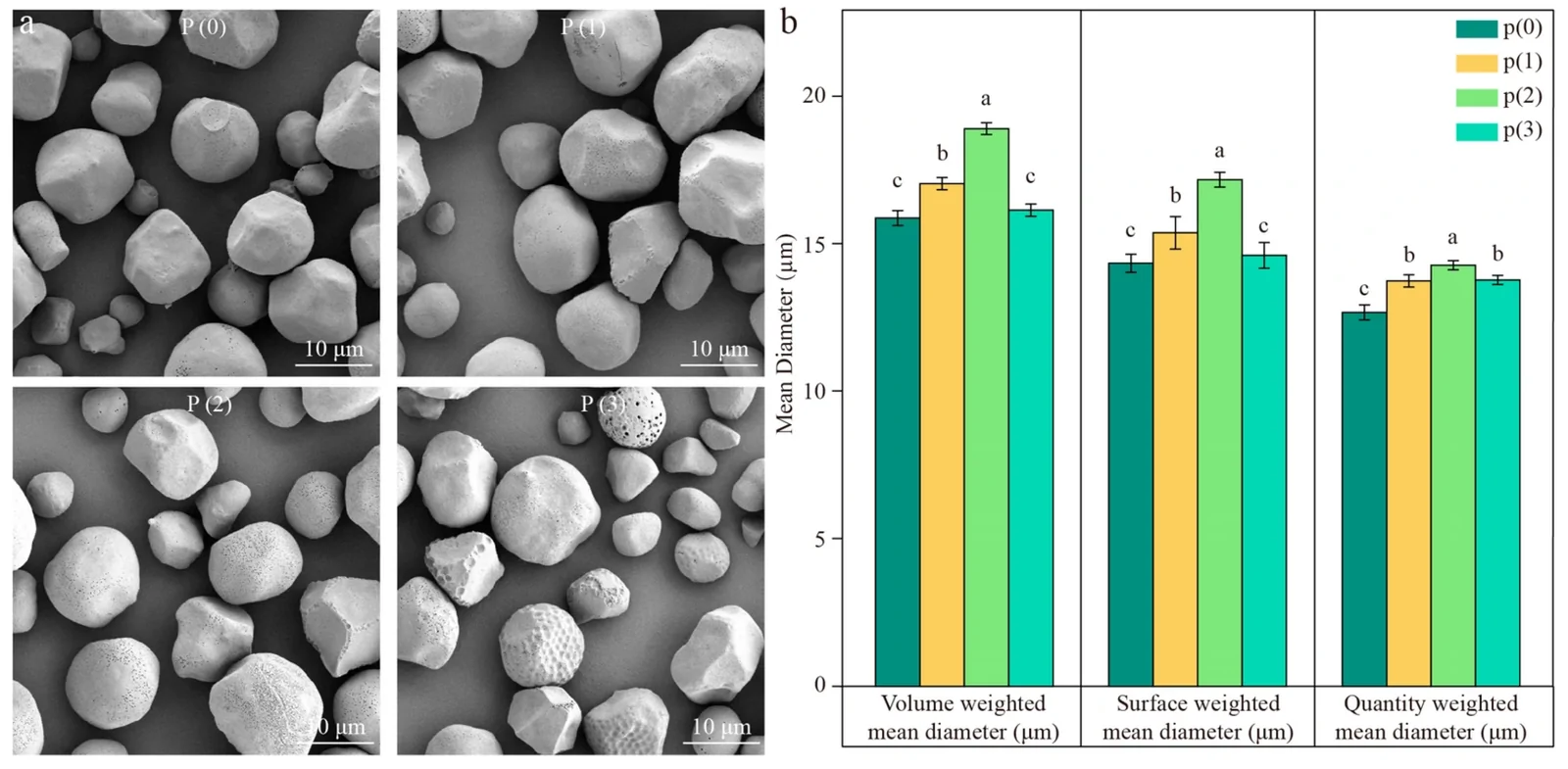
Starch Granule Morphology and Size Distribution. (**a**) SEM images of starch from sorghum grains treated with different phosphorus levels. (**b**) Particle size distribution of sorghum starch under different phosphorus treatments. Different lowercase letters indicate statistically significant differences at *p* < 0.05.

In line with the phosphorus-induced variation in starch biosynthetic enzyme activities, notable differences in starch granule surface morphology were observed among phosphorus treatments. Scanning electron microscopy (SEM) showed that starch granules from all treatments generally exhibited irregular spherical or polygonal shapes. However, distinct surface alterations became apparent under the highest phosphorus treatment. Specifically, starch granules from the P3 treatment displayed prominent golf ball–like depressions and pit-shaped surface features, whereas granules from the P0 to P2 treatments maintained relatively smooth and compact surfaces. The formation of surface depressions under the highest phosphorus treatment may be associated with altered starch deposition patterns during grain filling. As demonstrated by enzyme activity analysis (Figure 3), moderate phosphorus input (P2) effectively enhanced the activities of AGPase, GBSS, SSS, and SBE, while further phosphorus addition (P3) failed to sustain this enhancement, particularly for GBSS. Such changes in enzyme activities may affect the coordination of glucan chain elongation and branching, potentially contributing to heterogeneous starch deposition within granules. Previous studies have shown that irregular organization of amylose and amylopectin can generate structurally unstable regions, which are prone to collapse or deformation and may ultimately manifest as surface depressions [8,25]. These results suggest that the highest phosphorus treatment, although not further increasing starch accumulation, may be associated with altered starch granule structural integrity through changes in starch deposition processes.

The analysis of starch granule size distribution after harvest demonstrated that phosphorus availability markedly influenced starch granule development in sorghum. Overall, starch granules across all treatments exhibited a mean diameter ranging from 12.4 to 19.1 μm. As phosphorus application increased from P0 to P2, the mean granule size gradually increased, indicating that adequate phosphorus supply was associated with enhanced granule growth and volumetric expansion during grain filling. In contrast, under the highest phosphorus treatment (P3), the average granule size declined, suggesting that further phosphorus application did not promote additional granule enlargement and was associated with reduced granule size. This response pattern may be related to phosphorus-induced changes in starch biosynthesis-related enzyme activities. Under low to moderate phosphorus conditions (P0–P2), enhanced activities of AGPase, GBSS, SSS, and SBE may contribute to more efficient starch deposition within endosperm cells, thereby supporting continuous starch deposition within endosperm cells and allowing granules to expand to larger final sizes [8,26]. By contrast, under the highest phosphorus treatment (P3), the stimulatory effects on starch-synthesizing enzymes—particularly GBSS—were diminished, which may affect the coordination of amylose and amylopectin deposition and thereby limit further starch granule expansion. Comparable inhibitory responses have been reported in other crops, where starch granule development is constrained under specific fertilization regimes, resulting in smaller mature granules in rice, foxtail millet, and buckwheat [21,27,28]. The highest phosphorus treatment may alter normal starch deposition dynamics, which could contribute to reduced granule growth or an increased proportion of smaller granules, ultimately decreasing the overall mean particle size. Collectively, these results indicate that phosphorus influenced starch granule size in a dose-dependent manner within the tested phosphorus range, whereas the highest application rate was associated with less favorable granule development, underscoring the importance of rational phosphorus management in starch quality formation.

### 3.4. Effects of Phosphorus Supply on Starch Molecular Weight

Phosphorus supply had a significant effect on starch molecular weight (Mn, Mw, Mz), with clear differences in molecular weight distribution observed among the phosphorus treatments (Table 1). These variations indicate that phosphorus availability affected not only the average molecular size of starch molecules but also the breadth and heterogeneity of their molecular weight distribution. Under the no-phosphorus treatment (P0), starch exhibited a smaller Mn of 6.07 × 107 g/mol, indicating a relatively low number-average molecular weight. At the same time, the dispersity index (PDI) was 1.89 and the root-mean-square radius (Rz) was 268.90 nm, reflecting the molecular-weight dispersity and molecular dimensions of starch, respectively. This pattern was accompanied by relatively lower activities of starch biosynthesis-related enzymes under P0, suggesting a possible association between enzyme activity and starch molecular characteristics [29]. As phosphorus supply increased from P1 to P2, the molecular weight of starch gradually increased, with Mn reaching 7.43 × 107 g/mol under P2. The PDI decreased slightly from 1.82 under P1 to 1.79 under P2, although the difference was not statistically significant. Rz under P2 was higher than that under P0, it remained lower than that under P1 and P3, indicating that phosphorus treatment also influenced the molecular dimensions of starch. These changes were consistent with the enhanced activities of starch biosynthesis-related enzymes such as AGPase and GBSS under P2 and may be associated with differences in glucan chain development and molecular organization [29,30]. However, under the highest phosphorus treatment supply (P3), Mn decreased to 6.52 × 10^7^ g mol^−1^, whereas Mw and Mz increased to the highest values among all treatments. Meanwhile, PDI and Rz also increased significantly. These results indicate that P3 produced a broader and more heterogeneous molecular weight distribution, together with larger molecular dimensions. The coexistence of lower-number-average molecules and larger high-molecular-weight fractions suggests an imbalance in glucan chain elongation, branching, and molecular assembly under the highest phosphorus treatment input [31]. In summary, phosphorus supply significantly affected starch molecular weight, molecular weight distribution, dispersity, and molecular dimensions. These molecular changes were accompanied by differences in starch biosynthesis-related enzyme activities, suggesting that phosphorus-induced changes in enzyme activity may be associated with the observed variation in starch molecular characteristics.

### 3.5. Effects of Phosphorus Supply on Starch Fine Structure

**Figure 4:**
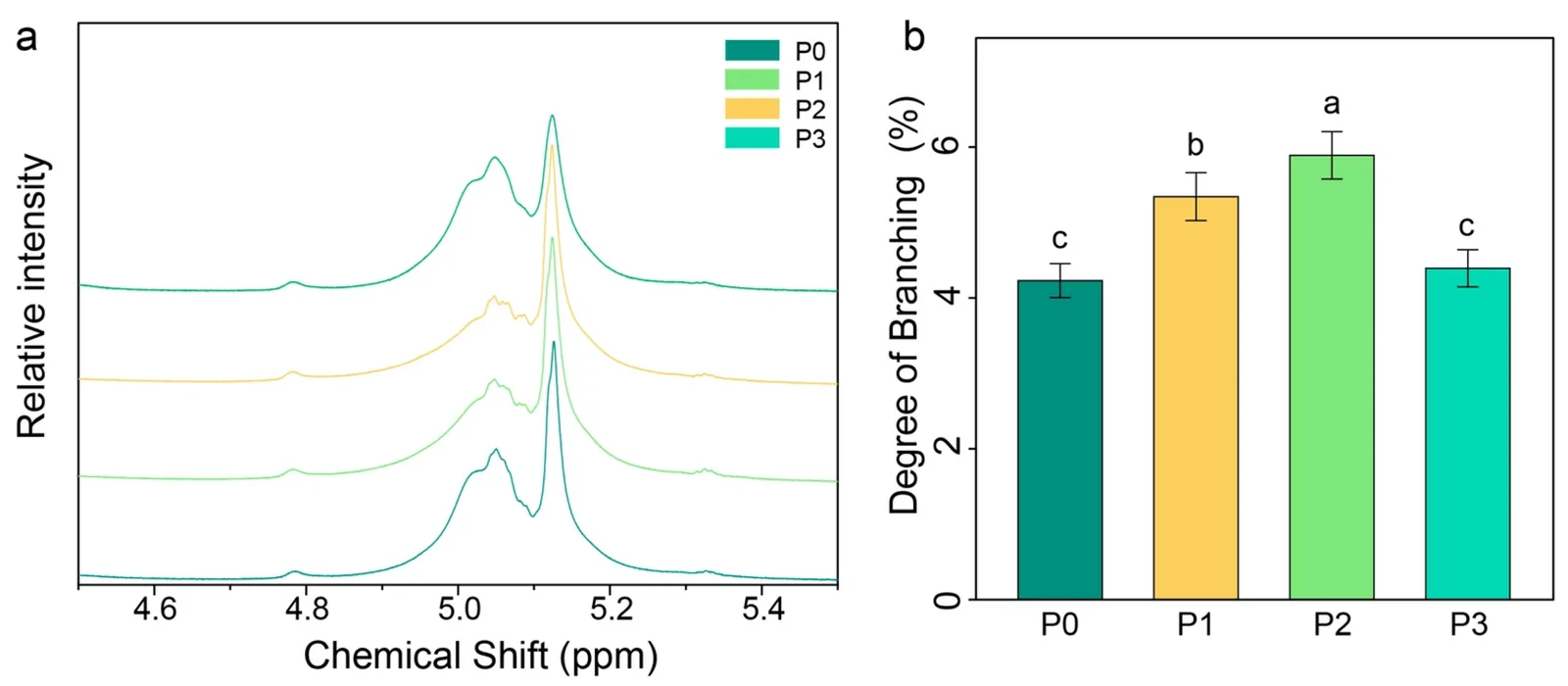
The effects of different phosphorus treatents on the degree of branching of sorghum grain starch. (**a**) Representative ^1^H NMR spectra of starch from different phosphorus treatments. (**b**) Degree of branching in starches under different phosphorus treatments. Different lowercase letters indicate statistically significant differences at *p* < 0.05.

Phosphorus fertilization significantly affected the starch chain length distribution, with clear differences observed among the various phosphorus treatments (Figure 4, Table S5). Under the no-phosphor, the proportion of short-chain amylopectin (A and B1 chains, DP 6–24) was higher, while the proportion of medium-chain (B2) and long-chain (B3) amylopectin was lower. As phosphorus supply increased from P1 to P2, the proportion of short-chain amylopectin slightly decreased, while the proportion of medium- and long-chain amylopectin gradually increased. Under P2 treatment, the proportion of B2 and B3 chains reached 14.34% and 12.32%, respectively. This pattern indicates that P2 was associated with a shift in amylopectin chain length distribution toward a greater relative abundance of medium- and long-chain fractions, which may be related to changes in starch biosynthesis-related enzyme activities [8]. In the case of the highest phosphorus treatment (P3), the proportion of long-chain amylopectin increased to 13.49%, despite a further decrease in short-chain amylopectin. These results indicate that P3 further altered the final amylopectin chain length distribution toward a higher relative abundance of long chains [12,32]. The observed changes in chain length distribution were accompanied by differences in the activities of starch biosynthesis-related enzymes (Figure 2), suggesting a possible association between enzyme activity and amylopectin structural organization.

Similarly, as phosphorus fertilization increased, the degree of branching continuously improved, peaking at 5.89% under P2 treatment (Figure 4b, Table S6). However, under P3, the branching degree decreased to 4.39 ± 0.20%, which was similar to that under P0. Together with the increase in the relative abundance of long-chain amylopectin, this result suggests that P3 was associated with a shift toward longer amylopectin chains but a lower degree of branching. These structural changes may be related to differences in starch biosynthesis-related enzyme activities. In conclusion, phosphorus fertilization significantly affected both amylopectin chain length distribution and branching degree.

### 3.6. Effects of Phosphorus Fertilization on Crystal Structure and Short-Range Molecular Order of Starch

**Figure 5:**
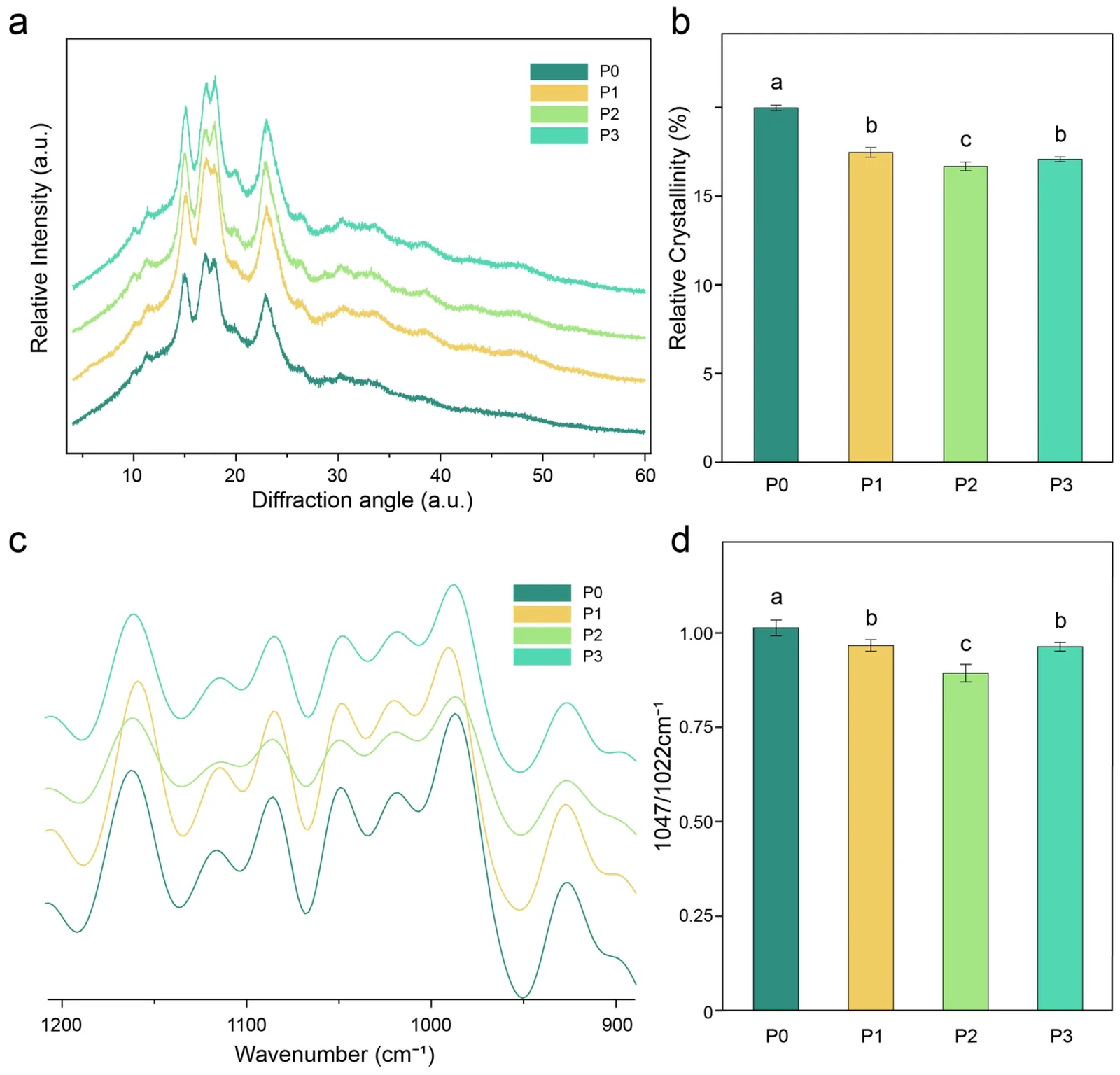
The effects of different phosphorus fertilizer treatments on the crystalline structure and molecular order of sorghum grain starch. (**a**) X-ray diffraction (XRD) patterns; (**b**) relative crystallinity; (**c**) Fourier-transform infrared (FTIR) spectra; (**d**) the 1047/1022 cm^−1^ absorbance ratio of sorghum starch. Different lowercase letters indicate statistically significant differences at *p* < 0.05.

Figure 5 shows the X-ray diffraction patterns of sorghum starches under different phosphorus treatments. All samples exhibited typical A-type crystalline characteristics, with diffraction peaks at 2θ = 15.1°, 17.2°, 17.9°, and 23.2°, but no visible peak at 2θ = 5.6°, indicating that phosphorus application did not alter the crystalline polymorph of sorghum starch. However, phosphorus application significantly affected relative crystallinity. Relative crystallinity decreased from P0 to P2, reaching the minimum value at P2 (16.68%), and then slightly increased at P3 (Table S7). Starch crystallinity is mainly associated with the ordered packing of double helices formed by amylopectin side chains, and can be influenced by amylose content and the arrangement of starch chains [12,33,34]. Therefore, the decrease in relative crystallinity from P0 to P2 may be related to changes in amylose content and starch-chain organization. The slight increase at P3 suggests a modest increase in crystalline order under the highest phosphorus treatment [35].

FTIR spectra were further used to evaluate the short-range molecular order of sorghum starch (Figure 5c). All samples showed similar absorption bands at 995, 1150, 1650, 2930, and 3380 cm^−1^. The bands near 995 and 1150 cm^−1^ were associated with C–O and C–C vibrations, respectively, while the band near 1650 cm^−1^ was attributed to the O–H bending vibration of absorbed or bound water. The bands near 2930 and 3380 cm^−1^ were associated with C–H and O–H stretching vibrations, respectively [36]. In the 1200–900 cm^−1^ region, characteristic bands near 1047 and 1022 cm^−1^ were identified. The 1047/1022 cm^−1^ ratio was used as an empirical indicator of short-range molecular order. As shown in Table S8, this ratio decreased by 11.85% in P2 compared with P0, indicating a reduction in short-range molecular order. It then increased slightly at P3, suggesting a partial recovery of local molecular order under the highest phosphorus treatment. Overall, phosphorus application did not change the A-type crystalline pattern of sorghum starch but affected its relative crystallinity and short-range molecular order. Both parameters decreased initially and then increased slightly at the highest phosphorus level, indicating that phosphorus supply influenced the multiscale structural organization of sorghum starch.

### 3.7. Effects of Phosphorus Treatment on Water Solubility and Swelling Power of Sorghum Starch

**Figure 6:**
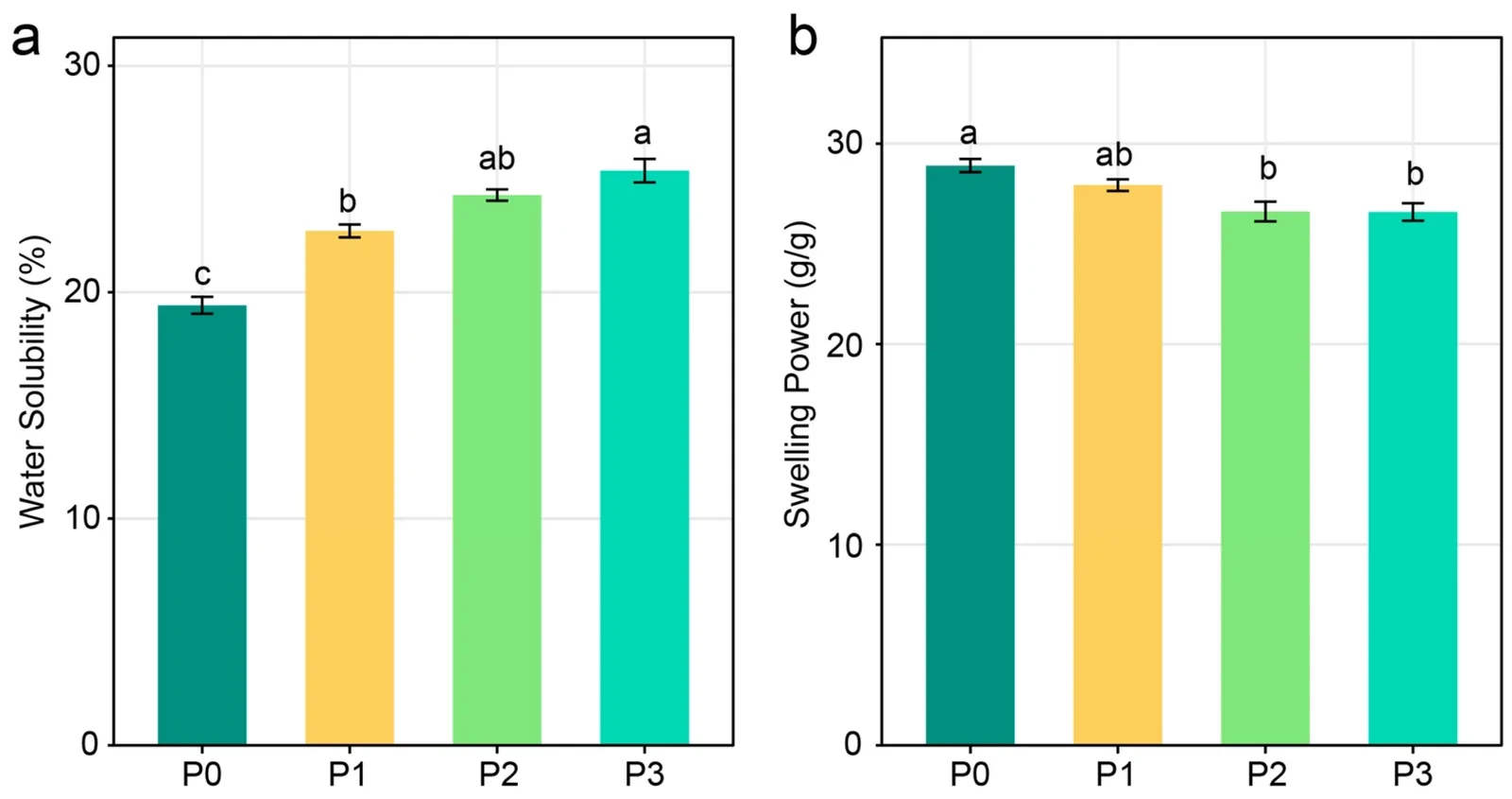
The effects of different phosphorus fertilizer treatments on the water solubility and swelling power of sorghum starch measured at 95 °C. (**a**) Water solubility (WS); (**b**) swelling power (SP). Different lowercase letters indicate statistically significant differences at *p* < 0.05.

Figure 6 shows the water solubility (WS) and swelling power (SP) of sorghum starch under different phosphorus treatments. Phosphorus application significantly affected the hydration properties of starch granules. Compared with P0 (19.42%), WS increased under phosphorus treatments, reaching the highest value of 25.36% at P3 (Table S9). In contrast, SP showed a decreasing trend with phosphorus application, with the lowest value observed at P3 (26.59 g/g). WS reflects the leaching of soluble starch components during heating, whereas SP represents the water absorption and swelling capacity of starch granules. The increase in WS suggests that phosphorus treatment promoted the dissolution of starch molecules from the granules. The increased WS may be associated with phosphorus-induced changes in starch molecular organization, including reduced crystalline order, altered amylose leaching, and modified chain interactions during heating [37]. However, the decrease in SP indicates that phosphorus treatment may restrict granule swelling, possibly due to changes in starch chain interactions within the granules. Similar results were reported in mung bean starch, where phosphorus fertilizer increased WS but reduced SP [8]. In addition, WS and SP are important indicators of the interactions among starch chains in amorphous and crystalline regions, and are affected by amylose and amylopectin contents, phosphorus level, granule size, and molecular conformation [38,39]. Therefore, appropriate phosphorus management may help regulate the hydration properties and processing suitability of sorghum starch. Sorghum starch with higher WS may be more suitable for products requiring rapid hydration, starch solubilization, and uniform paste formation during heating, whereas starch with higher SP may be beneficial for foods requiring greater water retention and softer texture [40,41].

### 3.8. Effects of Phosphorus Fertilizer on Pasting Properties of Sorghum Starch

The pasting properties of sorghum starch varied markedly among phosphorus treatments (Table 2). Compared with P0, phosphorus application generally increased peak viscosity (PV), breakdown viscosity (BD), final viscosity (FV), and setback viscosity (SB), whereas trough viscosity (TV) showed only minor variation among treatments. The highest PV and BD were observed under P3, reaching 1787.00 and 987.67 cP, respectively. FV reached its highest value under P2 (1535.33 cP), which was similar to that under P3 (1534.67 cP). SB was also highest under P2 (745.00 cP), followed closely by P3 (735.33 cP), and both values were significantly higher than that of P0. Peak time decreased from 4.48 min under P0 to 4.37 min under P3, indicating a shorter time required to reach peak viscosity at the highest phosphorus treatment.

The increase in PV under phosphorus fertilization indicates enhanced viscosity development during heating. However, this response did not parallel the independently measured swelling power, which decreased with phosphorus application, suggesting that the pasting behavior of sorghum starch was influenced by multiple structural factors rather than granule swelling alone. Changes in amylose content, molecular weight distribution, amylopectin chain length distribution, crystalline organization, and granule characteristics observed in this study may collectively contribute to the differences in viscosity development among treatments [42]. In particular, the relatively high PV under P3 coincided with a broader molecular weight distribution and a higher proportion of long amylopectin chains, indicating that changes in starch molecular organization may be involved in the altered pasting response.

BD increased markedly under phosphorus fertilization, particularly under P2 and P3, indicating a greater loss of viscosity after peak viscosity was reached during continued heating and shear. This suggests that the starch pastes formed under these treatments were less stable under thermal and mechanical stress despite their higher peak viscosities. In contrast, the higher FV observed under P2 and P3 indicates greater viscosity development during cooling. The accompanying increase in SB reflects a stronger tendency for starch molecules to reassociate as the paste cooled. This behavior may be related to the altered amylose content and molecular organization of starch, which influence intermolecular interactions during cooling [43].

Overall, phosphorus fertilization substantially modified the pasting behavior of sorghum starch. P2 was characterized by relatively high final and setback viscosities, whereas P3 produced the highest peak and breakdown viscosities together with a shorter peak time. These differences indicate that phosphorus application altered both viscosity development during heating and molecular reassociation during cooling, thereby affecting the processing characteristics of sorghum starch.

### 3.9. Effects of Phosphorus Fertilizer on Thermal Properties of Sorghum Starch

The thermal properties of sorghum starch under different phosphorus treatments are shown in Table S10. Starch thermal properties are commonly described by onset temperature (To), peak temperature (Tp), conclusion temperature (Tc), and gelatinization enthalpy (ΔH), which reflect the temperature and energy required for disrupting ordered structures during heating. Compared with P0, phosphorus application decreased To, Tp, and Tc from P0 to P2, followed by a slight increase at P3. The lowest To, Tp, and Tc were observed under P2 treatment, reaching 65.93 °C, 70.37 °C and 76.03 °C, respectively. These decreases indicate that P2 lowered the temperature required for starch gelatinization and disruption of ordered structures. Previous studies have shown that gelatinization temperature is closely related to the stability and integrity of starch crystalline regions [44,45]. Consistent with the gelatinization temperatures, ΔH also decreased from P0 to P2 and slightly increased at P3, with the lowest value observed under P2 treatment (13.10 J/g). ΔH represents the energy required to disrupt double-helical structures and crystalline regions during gelatinization. Therefore, the lower ΔH under P2 indicates a lower energy requirement for gelatinization and is consistent with reduced structural order in starch. This result agrees with the XRD and FTIR findings, where relative crystallinity and the 1047/1022 cm^−1^ ratio both decreased from P0 to P2 and partially recovered at P3. Previous studies also reported that ΔH is closely associated with starch crystallinity and double-helical stability [46,47]. The changes in thermal properties may also be related to starch hydration behavior. In this study, phosphorus application increased water solubility (WS) but decreased swelling power (SP), indicating enhanced starch molecule leaching and restricted granule swelling during heating. These changes may contribute to the lower temperature and energy requirements for disruption of ordered structures. Overall, phosphorus treatment altered the gelatinization behavior of sorghum starch, with P2 showing the lowest gelatinization temperatures and enthalpy, followed by a slight increase at P3. This suggests that phosphorus management can regulate the thermal behavior of sorghum starch by modifying its ordered structure and hydration characteristics.

### 3.10. Phosphorus Fertilization Regulates Starch Processing Properties Through Coordinated Structural Changes

**Figure 7:**
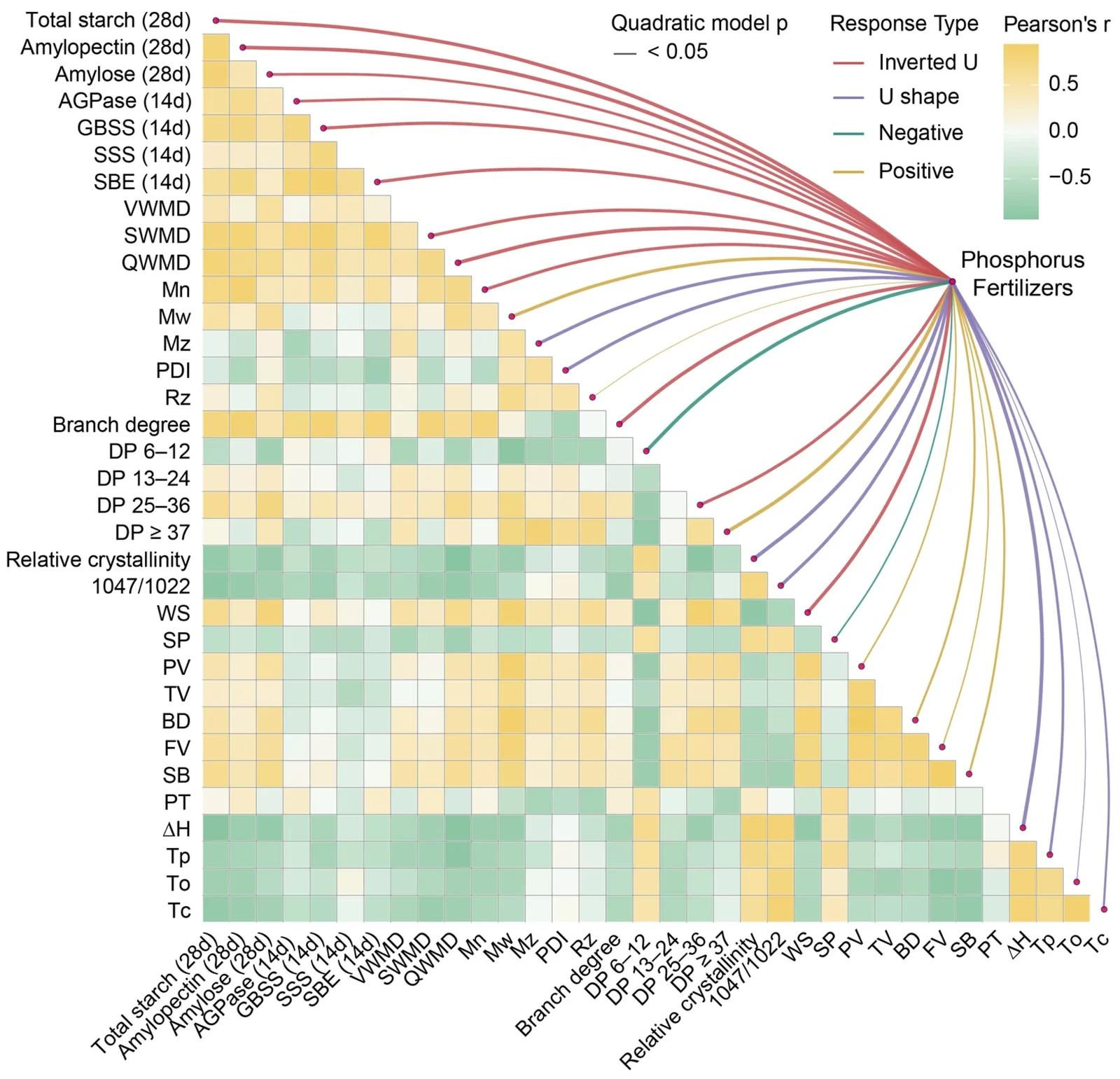
Correlations among starch composition, structural characteristics, and processing properties, together with exploratory fitted patterns across phosphorus fertilizer treatments. The lower-triangular heatmap shows Pearson correlation coefficients among the measured traits, with color and color intensity indicating the direction and strength of the correlations, respectively. Curved links between phosphorus fertilizer treatment and individual traits indicate exploratory fitted response patterns within the tested phosphorus range. Link colors denote apparent inverted-U-shaped, apparent U-shaped, positive linear, and negative linear patterns, whereas link width is proportional to the coefficient of determination (R^2^) of the fitted model. Because only four phosphorus application levels were tested, these fitted patterns should be interpreted as exploratory descriptions within the tested range rather than as robust or definitive biological dose–response relationships.

Correlation analysis indicated that starch processing performance was associated with coordinated variation in molecular architecture, granular organization, and crystalline order rather than with total starch accumulation alone (Figure 7, Table S11). After Benjamini–Hochberg correction, amylose remained positively associated with water solubility and setback viscosity, but negatively associated with relative crystallinity, the 1047/1022 cm^−1^ ratio, and gelatinization enthalpy. These results suggest that variation in amylose content was associated with both structural organization and selected processing properties. Because gelatinization involves the disruption of crystalline and short-range molecular order, reduced structural order may facilitate water penetration and molecular leaching [48,49]. Amylopectin chain length distribution also showed significant associations with starch functionality. B2 chains (DP 25–36) were positively associated with water solubility and breakdown viscosity, but negatively associated with crystallinity and gelatinization enthalpy. In contrast, A chains (DP 6–12) showed generally opposite associations with these structural and pasting properties. These patterns suggest that variation in amylopectin chain length distribution was related to differences in crystalline organization, hydration, and pasting behavior [42,50]. Granule size was likewise associated with starch structure and functionality. Quantity-weighted mean diameter was positively associated with water solubility, but negatively associated with swelling power, crystallinity, short-range molecular order, gelatinization enthalpy, and peak gelatinization temperature. Thus, larger granules in the present dataset were associated with lower structural order and thermal stability, indicating that granule size should be considered together with molecular composition and structural organization when interpreting starch functionality [51].

Exploratory regression analysis was used to describe several fitted response patterns within the tested phosphorus range of 0–160 kg P_2_O_5_ ha^−1^ (Table S12). Amylose, SWMD, QWMD, DP 25–36 chains, water solubility, total starch, amylopectin, and branching degree showed apparent inverted-U-shaped patterns, whereas relative crystallinity, the 1047/1022 cm^−1^ ratio, gelatinization enthalpy, and gelatinization temperatures showed apparent U-shaped patterns. In contrast, peak viscosity, breakdown viscosity, final viscosity, setback viscosity, Mw, and DP ≥ 37 chains showed positive linear trends within the tested range, while DP 6–12 chains and swelling power showed negative linear trends. Because only four phosphorus application levels were tested, these fitted relationships should be interpreted only as exploratory descriptions of the observed patterns within the tested range, rather than as robust or definitive biological dose–response relationships. A limitation of the present study is that phosphorus was supplied as single superphosphate, which also provided calcium and sulfur. Therefore, the observed responses cannot be attributed exclusively to phosphorus, and the potential contributions of accompanying calcium and sulfur should be considered. In addition, the experiment was conducted using one sorghum cultivar at a single location and during one growing season; thus, broader validation across cultivars, environments, and years is needed before making general agronomic recommendations.

## 4. Conclusions

This study showed that phosphorus fertilization was associated with significant changes in sorghum starch accumulation, structure, and functional properties. Changes in starch accumulation were accompanied by significant changes in the activities of starch biosynthesis-related enzymes, including AGPase, GBSS, SSS, and SBE. In particular, P2 showed relatively high enzyme activities and amylose content, suggesting an association between enzyme activity and starch accumulation and composition. Phosphorus fertilization was also associated with changes in starch granule size, molecular weight characteristics, branching degree, and amylopectin chain length distribution. P2 was characterized by a higher branching degree and greater relative proportions of medium- and long-chain amylopectin fractions. These structural changes were accompanied by lower relative crystallinity, short-range molecular order, gelatinization temperatures, and gelatinization enthalpy, indicating lower temperature and energy requirements for gelatinization. In contrast, the highest phosphorus treatment (P3) did not produce further enhancement in starch accumulation compared with P2 and showed lower activities of several starch biosynthesis-related enzymes. P3 was also associated with smaller granule size, greater molecular dispersity, and irregular surface depressions, suggesting differences in starch deposition and structural organization relative to P2. Functionally, phosphorus fertilization was associated with higher water solubility and pasting viscosity and lower swelling power and pasting time, reflecting coordinated differences in hydration, paste-forming properties, and granule stability. Correlation analysis showed that these functional changes were associated with coordinated changes in amylose content, amylopectin fine structure, granule morphology, and crystalline order.

Taken together, phosphorus fertilization was associated with coordinated changes in starch accumulation, molecular and granular structure, and functional properties. Within the phosphorus range investigated, P2 (80 kg P_2_O_5_ ha^−1^) was characterized by a combination of selected structural and thermal characteristics, including lower crystalline order and lower gelatinization temperature and enthalpy, whereas P3 produced the highest peak and breakdown viscosities. Thus, the appropriate phosphorus application rate may depend on the desired starch functionality and end-use requirements rather than representing a universal optimum. These results provide a useful basis for optimizing phosphorus management in relation to desired sorghum starch functionality and its potential applications in food processing and bio-based industries.

## Figures and Tables

**Table 1:** Molecular weight distribution and molecular size parameters of sorghum starch under different phosphorus treatments.

	Mn (×10^7^ g/mol)	Mw (×10^7^ g/mol)	Mz (×10^7^ g/mol)	PDI	Rz (nm)
P0	6.07 ± 0.13 c	11.45 ± 0.39 c	20.81 ± 0.13 b	1.89 ± 0.02 b	268.90 ± 10.41 b
P1	7.19 ± 0.34 ab	13.07 ± 0.41 b	20.31 ± 0.53 b	1.82 ± 0.03 b	306.82 ± 32.09 ab
P2	7.43 ± 0.32 a	13.26 ± 0.54 ab	21.17 ± 1.33 b	1.79 ± 0.00 b	291.55 ± 17.65 ab
P3	6.52 ± 0.32 bc	14.18 ± 0.27 a	25.83 ± 1.69 a	2.18 ± 0.15 a	341.93 ± 18.61 a

Note: Different lowercase letters (a,b,c) within the same column indicate significant differences (*p* < 0.05), whereas values sharing at least one common letter are not significantly different *(p* ≥ 0.05).

**Table 2:** Effects of different phosphorus fertilizer treatments on pasting properties of sorghum starch.

Treatment	PV (cP)	TV (cP)	BD (cP)	FV (cP)	SB (cP)	PT (min)
P0	1617.33 ± 14.66 b	775.67 ± 16.05 a	841.67 ± 10.21 b	1447.33 ± 31.48 b	671.67 ± 16.76 b	4.48 ± 0.02 a
P1	1715.67 ± 59.77 ab	785.67 ± 30.77 a	923.33 ± 45.39 ab	1488.67 ± 29.33 ab	696.33 ± 15.92 ab	4.44 ± 0.03 ab
P2	1726.67 ± 77.15 ab	790.33 ± 19.07 a	936.33 ± 58.09 ab	1535.33 ± 31.98 a	745.00 ± 15.64 a	4.47 ± 0.04 a
P3	1787.00 ± 43.30 a	797.33 ± 27.79 a	987.67 ± 33.51 a	1534.67 ± 43.71 a	735.33 ± 34.41 a	4.37 ± 0.04 b

Note: Different lowercase letters (a,b,c) within the same column indicate significant differences (*p* < 0.05), whereas values sharing at least one common letter are not significantly different (*p* ≥ 0.05).

## Data Availability

The original contributions presented in this study are included in the article/Supplementary Materials. Further inquiries can be directed to the corresponding authors.

## Supplementary Materials

The following supporting information can be downloaded at: https://www.mdpi.com/article/10.3390/foods15203651/s1, Table S1: Effects of phosphorus treatments on starch content and composition at different developmental stages, accounting for block effects; Table S2: Effects of phosphorus treatment, developmental stage, and their interaction on starch content and composition; Table S3: Effects of phosphorus treatment, developmental stage, and their interaction on starch biosynthesis-related enzyme activities; Table S4: Effects of phosphorus treatments on starch biosynthesis-related enzyme activities at different developmental stages, accounting for block effects; Table S5: Effects of different phosphorus fertilizer treatments on the amylopectin chain length distribution of sorghum starch; Table S6: Effects of different phosphorus fertilizer treatments on the degree of branching of sorghum starch; Table S7: Effects of different phosphorus fertilizer treatments on the relative crystallinity of sorghum starch; Table S8: Effects of different phosphorus fertilizer treatments on the 1047/1022 cm^−1^ absorbance ratio of sorghum starch; Table S9: Effects of different phosphorus fertilizer treatments on the water solubility and swelling power of sorghum starch; Table S10: Effects of different phosphorus fertilizer treatments on the thermal properties of sorghum starch; Table S11: Pairwise Pearson correlation analysis among measured variables based on plot-level data (*n* = 12), with Benjamini–Hochberg-adjusted *p*-values, and functional properties of sorghum starch; Table S12: Exploratory regression models describing starch-related responses to phosphorus application within the tested range.
