# Supplementary material for: From Phosphorus Nutrition to Starch Functionality: Effects of Phosphorus Fertilization on Sorghum Starch Biosynthesis, Multiscale Structure, and Processing Properties
Source: Foods. 2026 Oct 15;15(20):3651. doi: 10.3390/foods15203651 (PMC13703702; doi:10.3390/foods15203651)
Supplement: Supplementary file 1 [file foods-15-03651-s001.zip › Supplementary Tables S1-S10.pdf]

Table S1. Effects of phosphorus treatments on starch content and composition at different developmental stages, accounting for block effects.

Trait	P treatment	7 d	14 d	21 d	28 d
Amylopectin	P0	7.46 ± 0.38^{c}	16.00 ± 0.52^{b}	38.11 ± 0.45^{bc}	48.69 ± 0.52^{c}
	P1	8.42 ± 0.23^{b}	16.67 ± 0.31^{b}	38.70 ± 0.68^{ab}	51.93 ± 0.54^{b}
	P2	8.92 ± 0.09^{ab}	18.83 ± 0.35^{a}	39.55 ± 0.45^{a}	53.77 ± 0.73^{a}
	P3	9.63 ± 0.54^{a}	18.64 ± 0.79^{a}	37.44 ± 0.64^{c}	49.19 ± 0.34^{c}
Amylose	P0	2.96 ± 0.18^{a}	4.14 ± 1.08^{b}	11.86 ± 0.62^{c}	13.84 ± 1.98^{c}
	P1	3.19 ± 0.21^{a}	5.64 ± 0.34^{a}	13.41 ± 0.55^{b}	16.21 ± 1.00^{b}
	P2	2.88 ± 0.27^{a}	6.03 ± 0.30^{a}	16.17 ± 0.96^{a}	18.73 ± 1.05^{a}
	P3	2.89 ± 0.17^{a}	6.37 ± 0.38^{a}	12.50 ± 0.34^{bc}	17.81 ± 1.41^{ab}
Total starch	P0	10.41 ± 0.27^{c}	20.14 ± 0.70^{c}	49.97 ± 0.34^{c}	62.53 ± 1.76^{c}
	P1	11.61 ± 0.31^{b}	22.30 ± 0.64^{b}	52.12 ± 0.54^{b}	68.14 ± 0.90^{b}
	P2	11.81 ± 0.35^{ab}	24.86 ± 0.65^{a}	55.72 ± 1.12^{a}	72.50 ± 1.00^{a}
	P3	12.52 ± 0.40^{a}	25.01 ± 0.48^{a}	49.94 ± 0.32^{c}	67.00 ± 1.47^{b}

Note: Values are presented as mean ± SD of three independent field blocks (n = 3). Within each trait and developmental stage, phosphorus treatment effects were analyzed using an RCBD model including phosphorus treatment and block. Different lowercase superscript letters within the same column and trait indicate significant differences among phosphorus treatments according to Duncan's multiple range test at $P < 0.05$.

Table S2. Effects of phosphorus treatment, developmental stage, their interaction, and block on starch content and composition.

Trait	P effect	Stage effect	P × Stage	Block effect
Total starch	F = 88.03, P < 0.001	F = 11724.74, P < 0.001	F = 15.43, P < 0.001	F = 1.36, P = 0.272
Amylopectin	F = 57.58, P < 0.001	F = 17418.92, P < 0.001	F = 17.28, P < 0.001	F = 0.83, P = 0.444
Amylose	F = 21.61, P < 0.001	F = 699.70, P < 0.001	F= 5.63, P < 0.001	F = 1.16, P = 0.329

Note: Data were analyzed using an analysis of variance model including phosphorus treatment (P), developmental stage (Stage), the P × Stage interaction, and block. The experiment comprised three field blocks, with each phosphorus treatment represented once within each block (n = 3). Block was included in the model to account for the randomized complete block design. F values and corresponding P values are presented.

Table S3. Effects of phosphorus treatment, developmental stage, their interaction, and block on starch-biosynthesis-related enzyme activities.

Enzyme	Source	df	F-value	P-value
AGPase	Phosphorus treatment	3	21.572	<0.001
AGPase	Developmental stage	3	632.358	<0.001
AGPase	P × Stage	9	1.534	0.1814
AGPase	Block	2	0.413	0.6656
GBSS	Phosphorus treatment	3	31.304	<0.001
GBSS	Developmental stage	3	43.289	<0.001
GBSS	P × Stage	9	1.022	0.4457
GBSS	Block	2	0.776	0.4691
SSS	Phosphorus treatment	3	12.196	<0.001
SSS	Developmental stage	3	86.215	<0.001
SSS	P × Stage	9	0.786	0.6307
SSS	Block	2	0.998	0.3805
SBE	Phosphorus treatment	3	33.238	<0.001
SBE	Developmental stage	3	50.674	<0.001
SBE	P × Stage	9	1.557	0.1736
SBE	Block	2	0.658	0.5250

Note: Data were analyzed using an analysis of variance model including phosphorus treatment (P), developmental stage (Stage), the P × Stage interaction, and block. The experiment comprised three blocks, with each phosphorus treatment represented once within each block (n = 3). Block was included in the model to account for the randomized complete block design. F values and corresponding P values are presented.

Table S4. Effects of phosphorus treatments on starch-biosynthesis-related enzyme activities at different developmental stages, accounting for block effects.

Enzyme	P treatment	7 d	14 d	21 d	28 d
AGPase	P0	3.04 ± 0.06^{a}	5.00 ± 0.16^{bc}	2.43 ± 0.26^{b}	1.34 ± 0.10^{b}
	P1	3.12 ± 0.16^{a}	5.37 ± 0.23^{ab}	2.62 ± 0.37^{b}	1.53 ± 0.17^{b}
	P2	3.23 ± 0.20^{a}	5.62 ± 0.35^{a}	3.15 ± 0.19^{a}	2.06 ± 0.13^{a}
	P3	3.07 ± 0.08^{a}	4.81 ± 0.24^{c}	2.39 ± 0.14^{b}	1.29 ± 0.17^{b}
GBSS	P0	16.07 ± 1.05^{a}	18.70 ± 1.70^{d}	17.17 ± 0.95^{b}	14.37 ± 1.30^{b}
	P1	18.10 ± 1.15^{a}	22.50 ± 1.50^{b}	19.97 ± 1.60^{b}	16.23 ± 1.30^{ab}
	P2	19.23 ± 1.40^{a}	25.53 ± 1.70^{a}	23.33 ± 1.70^{a}	18.37 ± 1.26^{a}
	P3	16.93 ± 1.40^{a}	20.23 ± 1.21^{c}	18.67 ± 1.26^{b}	13.90 ± 1.10^{b}
SSS	P0	12.13 ± 0.75^{a}	18.20 ± 1.25^{c}	16.80 ± 1.25^{a}	11.27 ± 1.05^{b}
	P1	13.93 ± 1.15^{a}	19.30 ± 1.35^{b}	18.17 ± 1.50^{a}	12.33 ± 1.15^{ab}
	P2	14.37 ± 1.11^{a}	20.03 ± 1.80^{a}	19.20 ± 1.60^{a}	13.90 ± 1.00^{a}
	P3	12.13 ± 1.01^{a}	18.93 ± 1.85^{b}	15.07 ± 1.05^{a}	10.03 ± 0.90^{b}
SBE	P0	16.13 ± 1.15^{a}	19.83 ± 1.55^{c}	16.73 ± 1.20^{a}	15.13 ± 0.85^{a}
	P1	17.70 ± 1.21^{a}	22.87 ± 1.75^{b}	18.00 ± 1.25^{a}	16.33 ± 1.20^{a}
	P2	18.80 ± 1.45^{a}	25.93 ± 1.35^{a}	19.57 ± 1.65^{a}	17.53 ± 1.27^{a}
	P3	14.90 ± 1.21^{a}	18.20 ± 1.45^{d}	16.10 ± 1.20^{a}	12.03 ± 1.10^{b}

Note: Values are presented as mean ± SD of three field blocks (n = 3). Within each enzyme and developmental stage, phosphorus treatment effects were analyzed using a randomized complete block design model including phosphorus treatment and block. Different lowercase superscript letters within the same developmental stage indicate significant differences among phosphorus treatments according to Duncan's multiple range test at $P < 0.05$. When the overall phosphorus treatment effect within a developmental stage was not significant, all treatments were assigned the same letter.

Table S5. Effects of different phosphorus fertilizer treatments on the amylopectin chain-length distribution of sorghum starch.

Treatment	DP 6-12 (%)	DP 13-24 (%)	DP 25-36 (%)	DP ≥ 37 (%)
P0	25.36±0.36a	49.19±0.59a	13.52±0.23b	11.93±0.04c
P1	24.45±0.16b	49.03±0.43a	14.26±0.09a	12.26±0.23bc
P2	23.66±0.22c	49.68±0.25a	14.34±0.11a	12.32±0.08b
P3	22.50±0.41d	49.51±0.47a	14.50±0.20a	13.49±0.20a

Note: Values are presented as mean ± SD of three independent biological field replicates (n = 3). Different lowercase letters within the same row indicate significant differences among phosphorus treatments at $P < 0.05$ according to Duncan's multiple range test.

Table S6. Effects of different phosphorus fertilizer treatments on the degree of branching of sorghum starch.

Treatment	Degree of Branching (%)
P0	4.23±0.18c
P1	5.34±0.26b
P2	5.89±0.26a
P3	4.39±0.20c

Note: Values are presented as mean ± SD of three independent biological field replicates (n = 3). Different lowercase letters indicate significant differences among phosphorus treatments at P < 0.05 according to Duncan's multiple range test.

Table S7. Effects of different phosphorus fertilizer treatments on the relative crystallinity of sorghum starch.

Treatment	Relative Crystallinity (%)
P0	19.98±0.15a
P1	17.47±0.27b
P2	16.68±0.25c
P3	17.08±0.13b

Note: Values are presented as mean ± SD of three independent biological field replicates (n = 3). Different lowercase letters indicate significant differences among phosphorus treatments at P < 0.05 according to Duncan's multiple range test.

Table S8. Effects of different phosphorus fertilizer treatments on the 1047/1022 cm^{-1} absorbance ratio of sorghum starch.

Treatment	1047/1022 cm^{-1}
P0	1.013±0.021a
P1	0.967±0.015b
P2	0.893±0.023c
P3	0.963±0.011b

Note: Values are presented as mean ± SD of three independent biological field replicates (n = 3). Different lowercase letters indicate significant differences among phosphorus treatments at P < 0.05 according to Duncan's multiple range test.

Table S9. Effects of different phosphorus fertilizer treatments on the water solubility and swelling power of sorghum starch.

Treatment	Water Solubility (%)	Swelling Power (g/g)
P0	19.42±0.84c	28.91±0.73a
P1	22.71±0.64b	27.94±0.65ab
P2	24.29±0.56ab	26.62±1.11b
P3	25.36±1.16a	26.59±0.97b

Note: Values are presented as mean ± SD of three independent biological field replicates (n = 3). Different lowercase letters within the same row indicate significant differences among phosphorus treatments at P < 0.05 according to Duncan's multiple range test.

Table S10. Effects of different phosphorus fertilizer treatments on the thermal properties of sorghum starch.

Treatment	ΔH(J/g)	Tp (°C)	To (°C)	Tc (°C)
P0	16.75±0.18a	76.93±1.52a	70.83±1.96a	82.37±1.07a
P1	14.80±0.30b	74.30±1.61ab	69.23±0.96ab	81.30±0.67a
P2	13.10±0.42c	70.37±1.32c	65.93±1.60b	76.03±0.78b
P3	14.33±0.28b	72.97±1.41bc	68.67±1.23ab	80.13±1.43a

Note: Values are presented as mean ± SD of three independent biological field replicates (n = 3). Different lowercase letters within the same row indicate significant differences among phosphorus treatments at $P < 0.05$ according to Duncan's multiple range test.
